# Pupils’ Attitudes toward Inclusive Education

**DOI:** 10.3390/children10111787

**Published:** 2023-11-06

**Authors:** Anna Strnadova, Milon Potmesil, Petra Potmesilova

**Affiliations:** 1 Institute of Special Education Studies, Faculty of Education, Palacký University, 77900 Olomouc, Czech Republic; anna.strnadova01@upol.cz; 2 The Institute of Special Education Studies, Center of Evidence-Based Education & Arts Therapies: A Joanna Briggs Institute Affiliated Group, Faculty of Education, Palacky University, 77900 Olomouc, Czech Republic; 3 Department of Christian Education, St. Cyril and Methodius Faculty of Theology, Palacký University, 77900 Olomouc, Czech Republic; petra.potmesilova@upol.cz

**Keywords:** pupil with disabilities, non-disabled pupil, educational program, the concept of disability, society, attitudes

## Abstract

The presented research is a study of the attitudes of adolescents toward individuals with disabilities and a description of the level of knowledge about this issue among a selected group of adolescents. The study is focused on the school social environment, specifically on pupils without disabilities and their attitudes toward pupils with disabilities, which is one of the factors influencing their school success. The target group was 1806 pupils between the ages of 11 and 16 from the entire Czech Republic, approached by random selection. The research tool for the quantitative approach was a questionnaire developed by the authors of this article. The questionnaire section with open questions served for the qualitative part of the research. The results show that 70% of the respondents have basic knowledge about people with disabilities. Furthermore, it was shown that these adolescents have an overall positive attitude toward people with disabilities. This attitude is statistically significantly better among the girls than the boys, and the girls are also more open to discussing individuals with disabilities. At the same time, even if attitudes are positive, they still depend on the type of disability. The greatest uncertainty or discomfort is manifested when interacting with individuals with intellectual disabilities.

## 1. Introduction

Currently, when inclusive education is widespread in most educational systems, including the Czech Republic, research on the attitudes of adolescents toward disabled individuals, emphasizing key findings, trends, and gaps in the knowledge, is a significant contribution to theory and practice. Adolescent attitudes have their own developmental dynamics, just as the development of the reality of inclusive education shows elements of development over time [1]. There is a need to describe differences in adolescent attitudes toward disability, providing insights into factors that influence these attitudes, such as cultural values, education, and personal experiences. The impact of media portrayals on perceptions of disability and adolescent attitudes toward disabled individuals is also significant, with a need for discussion of both positive and negative portrayals and their potential consequences [2]. As far as the school environment is concerned, it is especially expected here to work with attitudes toward persons with disabilities and their education [3,4]. The results of research in this area should be used for policies and initiatives aimed at promoting equality, accessibility, and inclusion for people with disabilities to ensure their rights and quality of life [5,6]. Studying adolescent attitudes toward disability can provide valuable data for predicting future societal trends, informing strategic planning, and resource allocation in areas such as education, healthcare, employment, and available infrastructure.

There is a gap in society’s perception and willingness to accept persons with disabilities in the school environment because of low levels of awareness and a lack of informed experience [7,8].

School inclusion has a very wide range of definitions, reflecting national perspectives on this way of education. In general, inclusive education can be characterized as the best way to respect the diversity of the student population with opportunities for modification of the material and social environment [9,10,11].

Inclusive education (in the Czech environment, the term “Joint Education” is used) can be traced back to basic principles that are very similar to those in other countries [12]. It is about striving and creating conditions for the involvement of all pupils and students in the educational process. In an inclusive learning environment, all pupils, regardless of health, social, cultural, or other disadvantages, learn together and teachers compensate for these disadvantages and special educational needs and use support measures. This includes modification of teaching methods, support by a teaching assistant, or, in some cases, support provided by a personal assistant. Inclusive education aims to educate all pupils and students together, to promote and put into practice tolerance of difference, and to maximize collaboration between pupils [13]. In addition to inclusive education options, there is also the possibility of educating children with special educational needs in special classes and schools.

With this in mind, this study aims to explore the attitudes of non-disabled pupils toward co-education with pupils with disabilities. The school environment, especially the social climate in the classroom, is a significant factor influencing the academic achievement of all students [14,15]. Children’s relationships in the classroom influence their motivation to learn, but also affect the teacher’s interest in the work and motivation to perform the teacher’s work [16,17].

In the Czech literature, there are no articles that deal with the relationship of non-disabled pupils to persons with disabilities in relation to inclusive education. The target topics are teacher support, support for pupils in inclusive environments, and teachers’ attitudes toward inclusive education.

For this reason, a research project was designed to provide the first information on adolescents’ attitudes toward individuals with disabilities and basic knowledge of them. The characterization of attitudes in this age group is very important for the creation of the social climate in the school classroom and the design of appropriate interventions led by a special pedagogue or psychologist.

The aim was to find out the current attitudes of pupils without disabilities toward classmates with any disability. It was necessary to find out the situation and to create a set of information for teachers of the previous educational stage to try to include topics and activities that would have a positive influence on the attitudes of pupils without disabilities toward classmates with disabilities. Children cannot be expected to acquire this information and competence in their own families.

### 1.1. Inclusive Education in the Czech Republic

Inclusive education in the Czech Republic is based on the national and international legislative framework. At the national level, the Education Act [18] defines the types and level of support provided for children, pupils, and students with special educational needs in Section 16, “Education of children, pupils, and students with special educational needs”. The support measures have five levels and are applied to education in an inclusive framework at the primary and secondary levels, depending on the level of the pupil’s needs. Similarly, support is provided to students who need it at the university level. The current practice of education of children and pupils with special educational needs is regulated by Decree No. 27/2016 Coll. [19], on the education of pupils with special educational needs and gifted pupils, and its latest amendment (currently, the version from 1 January 2021 is in force; this is the eighth amendment). These documents detail all levels of support measures in terms of the content and form of support and also list the purpose of compensatory aids, special textbooks, and special teaching aids, their division into levels according to their use by the pupil, and the rules for their use by the school, including their standardized financial requirements. The support offered to inclusive education—to pupils and teachers—is characterized by Decree No. 72/2005 Coll. [20], on the provision of counseling services in schools and school counseling facilities, as amended (currently in force in the version from 1 January 2021—this is the sixth amendment). This describes the counseling facilities, institutions, and professions that are included in the counseling system used in inclusive education. These include diagnostic services, assistance in the construction of individual education plans, and advice for teachers concerning the necessary modifications to the educational process. This includes both methodological recommendations and counseling to make the necessary adjustments to the psychosocial climate in the classroom or school. In the Czech Republic, in the 2022/2023 school year, there were 3657 primary schools with pupils aged 6–15. In this period, there were 88,903 pupils with special educational needs. There were 84,344 pupils in inclusive education and 4559 pupils in special classes [21].

From the international perspective, the Convention on the Rights of the Child, which was negotiated on 20 November 1989 in New York [22], and entered into force under 104/1991 Coll., and the Convention on the Rights of Persons with Disabilities, negotiated on 13 December 2006 in New York [23], are very important. The Convention on the Rights of Persons with Disabilities, implemented in signatory countries since 2008, provides the basic framework for equality for persons with disabilities, guarantees the full enjoyment of all human rights, and promotes their active participation in society, which includes education.

The significance of these two fundamental documents can be summarized as a basic position on the rights of persons with disabilities, which includes the support provided, the protection offered, and the regulation of living conditions leading to equality, and, as regards all human rights and fundamental freedoms of persons with disabilities, also the promotion of respect for their inherent dignity in the same way as for non-disabled persons. These documents reflect a qualitative shift in the world’s response to disability in its understanding of disability and the possibilities for living life to the full.

The principles of inclusive education and equality of access to education are set out in the Czech national legislation. Attention is paid to persons with any kind of disability or disadvantage, following the conditions set out in particular in the Convention on the Rights of Persons with Disabilities (CRPD). The educational process is conceived as a dynamic interaction between the disabled pupil and the environment in a broader context that includes all participants and offers possibilities for modification so that a particular person with a disability can reach his or her full potential and fulfil the requirements of the educational program. Inclusive education is essentially built on four pillars: the pupil with special educational needs, the teacher, classmates, and parents. The fourth pillar, parents, is divided for some research purposes into two groups: parents of the pupil in inclusive education and parents of non-disabled pupils.

### 1.2. Inclusive Education and Stigma

Inclusive education is now an established educational format in many countries, reflecting the developmental dynamics of society. This is linked to both the successes of this approach and the barriers to it. The most common causes of difficulties are insufficiently prepared teachers, an insufficient level of special education support, the school climate with non-disabled pupils, and often parents of non-disabled pupils [24,25,26]. It should be noted that special education also features difficulties and barriers affecting success. These include teacher preparedness, stigmatization of children, or a lack of communication between the school and families [27,28].

The multilevel approach to stigma and public health, as described by Cook et al. [29], is a model that focuses on the complex and multidimensional nature of health-related stigma or health disparities. This approach recognizes that stigma can be triggered by a variety of factors such as cultural and structural issues, interpersonal interactions, and personal factors such as relationships and behaviors.

The stigmatization of people with disabilities is not a new issue and needs to be addressed. Stigma can start in the school environment, which is extremely competitive in all areas of a child’s life. This also includes the difference caused by the presence of a disability [30,31].

For education in inclusive settings, it is important to understand some stigma caused by disability/special educational needs as a significant factor influencing the access of individuals with disabilities to education in general and to their potential with an impact on aspirational levels.

The biopsychosocial perspective is a holistic approach that considers the biological, psychological, and social factors influencing an individual’s health and well-being. This perspective is particularly relevant in the field of education, where it can inform the understanding and support of students with functional disabilities. The International Classification of Functional Disabilities and Health (ICF) provides a framework for assessing and classifying these disabilities. It recognizes that disability is not solely determined by physical impairments but also by the interaction between impairments and environmental factors. Referencing the biopsychosocial perspective of the ICF in education allows for a comprehensive understanding of functional disabilities. By considering biological, psychological, and social factors when supporting students with disabilities, educators can create inclusive learning environments that promote optimal development [32].

These resources may vary depending on individual characteristics and the context. Overall, a multilevel approach to disability offers a comprehensive and systemic view that allows for the development of strategies to prevent and reduce the negative impact on the child, which can be reflected throughout the educational complex. The theoretical level is fundamental in the stigma model because it provides a basis for understanding and defining stigma. This model assumes that the stigmatized person is perceived and evaluated on the basis of various factors that are located on three dimensions: (1) the cognitive dimension—this dimension includes the beliefs and stereotypes that individuals have about the stigmatized person. For example, they may think that they are dangerous or incapable; (2) the emotional dimension—this dimension refers to individuals’ emotional reaction to the stigmatized person. For example, they may feel uncomfortable; (3) the behavioral dimension—this dimension refers to the behaviors and skills needed for social interaction. These three dimensions are interrelated and interactions between them can lead to stigma becoming more deeply embedded in individuals and society as a whole. Understanding these dimensions can help to reduce stigma and improve the lives of children who are educated in inclusive settings. For the educational setting, the tools that can be used to reduce the stigma against children with disabilities in mainstream schools are essential. On the basis of knowledge of how stigma works, strategies can be developed to combat it. These strategies can include public awareness and education, and support for specific students who are stigmatized [33].

### 1.3. The Aim of the Study

This study focuses on the third pillar in the above list—classmates who are aged 11–16. This is the period of puberty, which is often described as difficult for the individual experiencing it and those around them.

For this study, the period of puberty is characterized as a stage that is significant for the formation of attitudes toward environmental phenomena and persons in the school environment [34]. This environment, in the form of inclusive education, provides the space for the emergence of such a specific attitude as the relationship with the student with disabilities. The school environment introduces a very important element to this process, which is competence [35,36]. The high level of competition in school is another element that enters into the social relationships between children and also into the construction of attitudes—children try to conform to group dynamics while being left to themselves in the competition for the best personal results [37].

The attitudes of adolescents can be assessed as complex, consisting of cognitive, emotional, and conative components. During the adolescent period, attitudes tend to be changeable, malleable, and influential [38,39]. Socially desirable attitudes depend on the objective conditions of an individual’s upbringing and socialization process and the adolescent’s subjective relationship to the phenomenon. The tendency to radicalism that appears in this period is caused by the desire to have one’s own opinion and the tendency to reflect the group opinion of classmates, and this is framed by a lack of experience and a tendency to hypercriticism [40]. In this schema, one’s own opinion and attitudes are formulated in the school environment. Thus, attitudes toward minorities or differences often become a very fragile area for the construction and implementation of relationships between non-disabled members of the school class and a child with disabilities [2,41,42]. For this reason, the topic of relationships and the attitudes of non-disabled students toward a classmate with a disability was chosen for this study. When it comes to the relationship with a classmate with a disability, the determining factors are relationships and the environment [43].

Intervention programs can be used to educate and shape attitudes toward people with disabilities and seek to achieve attitudinal change based on information that reflects the reality of the school environment in which the disabled pupil is to be found [44].

Attitudes toward persons with disabilities are based on respect for differences, tolerance, and the ability to accept a classmate with a disability. Educational intervention should include encouragement to recognize and understand difference, education for empathy, and understanding of the specific needs that a disability brings to a student. In this way, the three basic areas on which the coexistence of the two “worlds” is based should be addressed. These are knowledge, skills, and emotional adjustment [45].

The research was focused on the age category of pupils (11–16 years old) who are in different grades in the Czech education system. The aim of the research was to obtain basic information about the attitudes of these pupils toward pupils with disabilities.

On the basis of the research objective, two main research questions and four “sub-questions” were identified:

I. What are adolescents’ attitudes toward individuals with disabilities?
II. What knowledge do adolescents have about individuals with disabilities?
  1. Does the respondent’s gender affect their attitude toward individuals with disabilities?
  2. Does the age of the respondent affect their attitude toward individuals with disabilities?
  3. Does a previous encounter with an individual with a disability affect their attitudes toward individuals with disabilities?
  4. Does adolescents’ knowledge about individuals with disabilities relate to their attitudes toward individuals with disabilities?

The following hypotheses were then established for the research questions:

**H1.**  *There is no statistically significant relationship between the respondent’s gender and their attitudes toward individuals with disabilities.*

**H2.**  *There is no statistically significant relationship between the age of the respondent and their attitudes toward individuals with disabilities.*

**H3.**  *There is no statistically significant relationship between a previous encounter with an individual with a disability and attitudes toward individuals with disabilities:*

*(a)*  *Presence of the individual in the family*
*(b)*  *A friend with a disability*
*(c)*  *Any encounter*

**H4.**  *There is no statistically significant relationship between knowledge about individuals with disabilities and attitudes toward individuals with disabilities.*

The hypotheses were constructed to describe the existing relationship between the respondent’s attitude and his/her age and gender. In planning the research, the results in these areas were important for recommending targeted interventions. The items concerning previous experience and the level of awareness were of similar importance. These findings should also influence the form of the planned intervention.

Teachers of pupils aged 6–11 in the first stage of primary school play a key role in how children perceive the world around them. Teachers need to teach children about a variety of topics, including those related to disability. One of the most important tasks for teachers in Key Stage 1 is to teach children how to behave and interact appropriately with people with different types of disabilities. It is important to stress that everyone has the right to equal respect and dignity and that everyone should be respected regardless of whether or not they have a disability. Teachers should also teach children about ways to cope with disability and ways in which disabled people can be supported. Learning about how to seek help, for example with special aids, so that people with disabilities can be as independent as possible, and linking them to organizations that help and support people with disabilities, can be very useful for children. For example, it may be possible to invite people with disabilities into the classroom to present their lives and answer children’s questions. Children can learn simple ways to help people with disabilities in their daily lives. Disability education should be part of everyday learning in the first year of primary school. This learning can also contribute to reducing the stigma against people with disabilities and creating better conditions for their inclusion in society. In addition to creating a haven for children with different types of disabilities in particular, and equipping them with the skills and knowledge needed for future independence, primary school teachers can also transform children’s behavior and their relationship to disability through their work. When today’s children grow up in inclusive classrooms, they feel more comfortable working with people with different kinds of disabilities, which can lead to a future society that is broader and more open to the different needs and experiences of people with disabilities. Disability education at the first level of primary school is therefore not only important for creating an inclusive society but also for developing empathy and respect among children. It is one of the basic tasks of teachers to teach children how to behave properly toward people with disabilities.

## 2. Materials and Methods

### 2.1. Participants

A total of 1806 respondents—students of secondary schools (*n* = 1806)—were able to respond (for more information, see Table 1). The mean age of the respondents was 13 and the modus and median age were also 13.

The aim of the research was to explore the attitudes of adolescents aged 11–16 toward individuals with disabilities and their basic knowledge of individuals with disabilities. Research into the attitudes of 11–16-year-olds is important for several reasons. The first reason is that this age group is very sensitive to changes in their environment and their opinions and attitudes are quickly formed. It is therefore important to understand what influences them in order to work with them. Research into the attitudes of the 11–16 age group can also help us to identify the needs and problems of this group. For example, if we find that young people have inappropriate attitudes toward a particular topic, we can begin to work on changing these attitudes through educational programs, psychological interventions, and so on. Overall, then, research on attitudes in this age group is very important for understanding this group of children and their needs. The information obtained can be used to design appropriate interventions led by a special pedagogue or psychologist. There are 14 regions in the Czech Republic. Three different primary schools from each region were randomly selected and contacted, making a total of 42 schools. In the last phase of the data collection, 14 schools signed up to participate—one school from each region.

### 2.2. Design

A mixed design was chosen for the research. A questionnaire and a semi-structured interview were used to collect data.

#### 2.2.1. Measurement

A questionnaire which had a total of 19 items was chosen for the data collection. The first 2 items were questions on demographic data (age, gender), while the remaining 17 items were related to the issue under study. The questionnaire was designed by the authors of this paper on the basis of a questionnaire constructed by [1], which had 12 items, each in the form of a statement to which respondents were asked to select a response in the form of a value on a scale from 1–5, where 1 indicated total agreement with the statement, 2 agreement with the statement, 3 a neutral attitude toward the statement, 4 some disagreement, and 5 total disagreement. All 12 of these items were used to construct the questionnaire for the research presented here.

The questions of the questionnaire were constructed to obtain data to assess the respondents’ attitudes in the area of three factors (acceptance, rejection–competence–opportunity), which emerged from the factor analysis. The interview to obtain qualitative data was conducted following the protocol [46].

In translating the items into English, we followed the Standards for Educational and Psychological Testing [47]. The items were translated by an independent translation service, then submitted for consultation with two different special pedagogues and one psychologist. The proposed modifications were consulted with the submitter and a pilot version of the 12 items was created. These items were presented to 15 respondents aged 11–16 years. Suggestions for revisions of the items were again consulted with the translator, and a final version of the 12 original items in the Czech language was created.

All the experts who participated found no inconsistencies in the translations of the instrument. The individual items of the original questionnaire were clearly worded, which made the translation easy and understandable.

The remaining items (four items) were created by the authors of the questionnaire following the research objectives and were again subjected to consultation by two special pedagogues and one psychologist. In this case, too, piloting was carried out with 15 respondents. The last item was left as an opportunity to say anything on the topic. These five items explored the respondents’ experiences with persons with disabilities in their own family and social environment. The respondents’ awareness was determined by completing a task in which respondents were asked to create pairs of terms that belonged together. The responses from these items were then correlated with items from the original questionnaire.

The questionnaire was distributed electronically. Principals of schools across the country educating adolescents aged 11–16 years were contacted. The questionnaire was administered during school hours. Teachers were instructed not to help or influence the adolescents. It was then a random selection within the chosen age range.

In the course of contacting schools to ask them to complete the questionnaires, a request was also made to participate in a semi-structured interview. A total of ten respondents answered this call. The interviews were conducted in person. To preserve anonymity, only these ten respondents were contacted to obtain qualitative data in interviews, a sufficient number for this method. The data collection took place from April to November 2022.

#### 2.2.2. Data Processing

The data were fixed in Microsoft Excel 2016; statistical processing was performed in Microsoft Excel and IBM SPSS 29 (independent-samples t-test, chi-square, and the Pearson correlation coefficient). The last question in the questionnaire was open-ended and was then assessed qualitatively using open coding. The interviews were again analyzed using open coding.

## 3. Results

The questions explored the respondents’ experiences with persons with disabilities in their own family and social environment were evaluated first (see Table 2).

For all three questions, in the case of an affirmative answer, the type of disability was also collected. In the first case, intellectual disability was the most represented (4.6%), while in the second and third cases, multiple disabilities were the most represented (11% and 35% respectively).

It should be added that the term “don’t know” included all the responses from respondents who wrote that they did not know, were not sure, or might have had experience but did not know what type or kind of disability it was.

The primary question of this research was adolescents’ attitudes toward individuals with disabilities. There was a total of 12 questions related to this issue, all of which took the form of a statement. For all the questions, the respondents used all the values of the scale. Basic statistical values (means, standard deviation, minima, and maxima) were calculated for all items.

The mean value for 9 out of the 12 questions was 3. The detailed results show that for six questions, the responses are distributed in such a way that about 40% are neutral responses and the remaining 60% are evenly split between agreeing and disagreeing with the statement. These questions are:

7. If I had a family member with a disability, would I tell my friends?
8. I feel comfortable in the company of people with disabilities.
9. I would go to a place where people with disabilities could be.
12. People with disabilities act like children.
14. Individuals with disabilities have a circle of friends similar to mine.
18. An unemployed person with a disability should have priority in employment over an able-bodied person.

For Question 18, although there is a slight tendency toward disagreeing answers, it is not as great as for the other questions. The frequencies and percentages of each response for the questions are shown in Table 3.

For the remaining six questions, the answers were always on one side of the scale. In the case of one question, this was a significant shift of the rating toward neutral to negative:

13. Children with disabilities should study in normal schools.

Only 21% of the respondents strongly agreed or agreed with this statement. In contrast, almost 50% disagreed or strongly disagreed with the statement.

For the other questions, it was the other way around—a tendency toward positive to neutral evaluations:

10. Interesting and varied work is suitable for individuals with disabilities.
11. If I am with an individual with a disability, I know how to treat them.
15. Individuals with disabilities should have the same opportunities in life as others.
16. Individuals with disabilities should live together with non-disabled people.
17. Individuals with disabilities can do both team and individual sports.

The strongest tendency toward positive evaluation was for Question 15 (Individuals with disabilities should have the same opportunities in life as others). Overwhelming agreement was elicited from 50% of the respondents. A total of 77% of the respondents included total agreement and agreement (see Table 4).

The second research question was: What is the adolescents’ knowledge about individuals with disabilities? There was one question related to this issue, which was divided into five sub-questions. The respondents were asked to connect two related concepts each time.

The adolescents have a basic understanding of each area. In the area of knowledge of physical disability, 95% answered correctly, while in the other areas the range was 86–88%. The respondents were expected to combine two concepts that are related to each other (e.g., surdopedia–sign language or typhlopedia–braille).

The author of the questionnaire [1] based the interpretation of the results on factor analysis. Three factors (acceptance, rejection–competence–opportunity) emerged from the factor analysis. In addition to the statistical methods mentioned below (t-test, chi-square, correlation), factor analysis was also chosen in this case. As a first step, the suitability of the data for factor analysis was examined. We calculated the Kaiser–Meyer–Olkin value (KMO) = 0.84 (*p* < 0.001), and an “antiimage matrix” was constructed in which the values of the measure of sampling adequacy (MSA) were greater than 0.5 for all items; more precisely, they ranged from 0.8 to 0.91. The final step involved determining commonality, i.e., the degree of correlation of an item with the other items entering the factor analysis. The commonality values for all items ranged from 0.4 to 0.6. The above data show that the data are suitable for factor analysis. The KMO value showed the average suitability of the data for factor analysis. The other values, MSA and commonality, confirmed the suitability of the items for factor analysis. We extracted three factors (see Table 5) that explained 46% of the variance in the items. The factors that were extracted did not correspond to the three scales in the original questionnaire. The principal component analysis method was used to extract the factors and the number of factors was selected on the basis of Kaiser’s rule. For easier interpretability, the factors were rotated using the varimax method.

The factors listed correspond to the different components of attitudes as defined as acceptance (cognitive), feelings (emotional), and competence (conative) in the introduction. Compared to the original questionnaire, the type and number of items that saturate each factor are different. However, the aim of this research was not to review the original questionnaire. Thus, we will continue to work with the results we obtained.

An independent samples *t*-test was used to evaluate Hypothesis H1. First, the hypothesis was tested in relation to all responses and then in relation to the individual factors. In the first case, there was a significant difference in the scores for boys (M = 2.8, SD = 0.54) and girls (M = 2.6, SD = 0.5) in terms of their attitudes toward individuals with disabilities; t (1790) = 6.67, *p* < 0.001. Using a sign scheme, it was then found that the girls have statistically significantly more positive attitudes toward individuals with disabilities. In the case of testing each factor, it was found that the null hypothesis could be rejected in the case of acceptance and feelings. On the other hand, in the case of competence, the null hypothesis must be accepted. Again, using the sign scheme, it was found that the girls show statistically significantly positive acceptance of individuals with disabilities and statistically significantly positive feelings toward individuals with disabilities:

Acceptance t (1790) = 4.91, *p* < 0.001

Feelings t (1790) = 7.42, *p* < 0.001

Competence t (1790) = 1.52, *p* = 0.065

On the basis of the results, we reject the null hypothesis and accept the alternative hypothesis. There is a statistically significant relationship between overall attitudes and gender and between acceptance and feelings and gender. However, this is not confirmed in the case of competence and the null hypothesis must be accepted.

A chi-square test of independence was used to test Hypothesis H2. The calculated values of χ^2^ (205, N = 1806) = 175.97, *p* = 0.93 indicate that the null hypothesis cannot be rejected. There is no statistically significant relationship in terms of age and overall attitudes toward individuals with disabilities. However, when one looks at individual factors, a statistically significant relationship with age cannot be confirmed in the case of acceptance and feelings. For competence, the opposite is true.

Acceptance χ^2^ (110, N = 1806) = 89.85, *p* = 0.92

Feelings χ^2^ (80, N = 1806) = 86.3, *p* = 0.3

Competence χ^2^ (40, N = 1806) = 98.8, *p* < 0.001

On the basis of the sign scheme, it was found that the eleven-year-olds were statistically significantly more likely to choose responses agreeing with the statements and the fifteen-year-olds were statistically significantly less likely to choose responses that agreed; that is, more of the eleven-year-olds reported knowing how to behave in the presence of individuals with special educational needs.

On the basis of these results, the null hypothesis cannot be rejected. There is no statistically significant relationship between overall attitudes toward individuals with disabilities and age and between acceptance and feelings and age. The null hypothesis can be rejected in the case of age and competence. There is a statistically significant relationship between age and competence.

The chi-square test of independence was used to test the hypotheses of H3. In the case of Hypothesis H3a (the presence of an individual in the family), the values of χ^2^ (82, N = 1806) = 185.81, *p* < 0.001 can indicate rejection of the null hypothesis and acceptance of the alternative hypothesis. For Hypothesis H3b (a friend with a disability), the χ^2^ values (82, N = 1806) = 104.17, *p* = 0.05 lead to rejection of the null hypothesis and acceptance of the alternative. And in the case of Hypothesis H3c (contact with an individual with a disability), the values of χ^2^ (82, N = 1806) = 113.72, *p* = 0.012 again lead to rejecting the null hypothesis and accepting the alternative.

The above suggests that there is a statistically significant relationship between personal experience with an individual with a disability and attitudes toward such individuals. Using the sign scheme and correlations, it was possible to find that previous personal contact influenced attitudes toward disability rather positively (see Table 6).

Next, the hypothesis was tested with respect to the individual factors.

Disability in the family

Acceptance χ^2^ (44, N = 1806) = 71.38, *p* = 0.006

Feelings χ^2^ (32, N = 1806) = 64.76, *p* < 0.001

Competence χ^2^ (16, N = 1806) = 25.89, *p* = 0.05

Friend with a disability

Acceptance χ^2^ (44, N = 1806) = 58.94, *p* = 0.07

Feelings χ^2^ (32, N = 1806) = 50.96, *p* = 0.01

Competence χ^2^ (16, N = 1806) = 10.69, *p* = 0.83

Effect of contact

Acceptance χ^2^ (44, N = 1806) = 83.15, *p* < 0.001

Feelings χ^2^ (32, N = 1806) = 66.84, *p* < 0.001

Competence χ^2^ (16, N = 1806) = 22.84, *p* = 0.12

Using correlations, it was possible to find that previous personal contact influenced attitudes toward disability rather positively (see Table 7). The sign scheme showed a statically significant tendency toward positive feelings for respondents who had previous contact (family, friend, or company) with an individual with a disability. Further, those who had no prior contact were significantly less likely to choose affirmative responses for the feelings factor.

In the case of the presence of a disability in the family, there is a statistically significant relationship to all factors. For the existence of a disabled friend, this statistically significant relationship exists only for feelings, and for contact with an individual with a disability, for acceptance and feelings.

There is a statistically significant relationship between prior experience of contact with an individual with a disability and overall attitudes toward individuals with disabilities—we accept the alternative hypothesis. For each factor, we reject the null hypothesis for the presence of an individual with a disability in the family. In the case of a friend with a disability, the null hypothesis can only be rejected for feelings, and for the last factor, for both acceptance and feelings.

The chi-square test of independence was chosen to test Hypothesis H4. The calculated values for all items χ^2^ (205, N = 1806) = 491.68, *p* < 0.001 indicate that the alternative hypothesis, i.e., that there is a statistically significant relationship between knowledge and attitudes, should be accepted. The sign scheme showed that those who have a significantly good level of knowledge have significantly better attitudes toward individuals with disabilities and, at the same time, those who have a low level of knowledge about the world of individuals with disabilities have significantly worse attitudes toward these individuals.

The possibility of rejecting the null hypothesis is then confirmed by the values for the individual factor domains:

Acceptance χ^2^ (110, N = 1806) = 225.54, *p* < 0.001

Feelings χ^2^ (80, N = 1806) = 177.65, *p* < 0.001

Competence χ^2^ (40, N = 1806) = 14.64, *p* < 0.001

Again, the sign schema showed that good knowledge of the world of people with disabilities leads to better overall perceptions of these individuals, whether in the areas of acceptance, feelings, or competence. The null hypothesis can be rejected for both overall attitude and individual factors.

The interviews were transcribed and analyzed using open coding. The following categories gradually emerged: regret, admiration, taboo, inclusion and segregation, differentiation

1. Pity
  - Disability is a very tragic thing.
  - I feel sorry for them; it must be hard to live with a disability.
  - It’s hard.
  - Because I find this topic sad even though I know we should talk about it but I feel sorry for these people.
2. Admiration
  - I would like to say that a lot of people shy away from disabled people and it’s a shame. People should know that they tend to be the nicest people. They should learn more about them and treat them like everyone else in life.
  - Disabled people are fine, I did the Swan Lake dance in primary school and it was an experience.
3. Taboo
  - Why did you have such sensitive questions here? Could anyone have been hurt or mentally harmed?
  - I don’t like to talk about this topic.
  - I don’t feel comfortable talking about it.
4. Inclusion and segregation
  - I think people with disabilities or other disabilities should have the same opportunities as non-disabled people but I would make schools separate because there is a higher risk of bullying.
  - I know from experience that some people with disabilities would be able to integrate into normal life quite easily, but most disabled people would not be able to get on properly and get along with people in ‘normal’ society.
  - We are learning more about this topic every day because one of my parents works in a special school and since I know how teaching is done in these schools, I think that being with qualified staff can help with inclusion among students with similar disabilities. However, pupils with physical disabilities should be taught to the same extent as able-bodied pupils.
5. Differentiation
  - Depending on the type of disability

The categories that emerged from the data collected in the interview corresponded with the categories that emerged from the analysis of Item 19 in the “opportunity for the expression” questionnaire.

## 4. Discussion

A total of 1806 respondents aged 11–16 years were approached to investigate adolescents’ knowledge and attitudes toward individuals with disabilities. Two primary research questions were asked:

## 5. What Are the Attitudes of Adolescents toward Individuals with Disabilities?

Statistical evaluation shows that the adolescents have rather positive attitudes toward individuals with disabilities. These attitudes are stronger among the girls than the boys. Similar conclusions were reached by Loeffler and Greitemeyer [48]. Different research conclusions were reached by Sharma, Pratap Yadav, and Sharma [49]. The results also showed that the girls are also more open to sharing information and attitudes. Using a sign scheme, the evidence was that if girls have someone with a disability in their family, they are significantly more likely to disclose this fact than boys are. Age no longer plays a role in these attitudes. However, on deeper examination, age was shown to play a role in relation to competence. The younger the individuals are, the more positive their view of individuals with disabilities. Looking more closely at the attitudinal assessment, the results show that, in general, adolescents have no problem meeting individuals with disabilities. Almost 70% of the respondents agree that individuals with disabilities should have equal opportunities, and almost 60% state that these individuals should live together with non-disabled individuals, i.e., they lean toward the idea of an inclusive society. The exception is the attitude toward co-education. There, on the contrary, almost 50% of the respondents reject this attitude. This finding among the sample of children in the present study is not consistent with the claim that has been made elsewhere that a positive attitude toward inclusive education is not a majority, but rather neutral with a preponderance of positive responses [50]. Here, a twofold explanation is offered; the first possibility is that it is an attitude where the individual generally agrees with inclusion but then in the case of personal space this is a problem. The answers to the questions that dealt with attitudes to the personal space (Q8 or Q9) then rather indicate that attitudes were normally distributed in the sample of respondents (about 30% rather agree or agree and 30% rather disagree or disagree). This means that it is not possible to say unequivocally how attuned the adolescents are to face-to-face encounters; it can only be stated that some are and some are not. Another reason may be that young people perceive social inclusion to be highly important. Thus, special education is not seen as segregating, but rather as a space in which to receive an education that is tailored to individuals with special educational needs. This education does not discriminate against them, but rather enables normal participation in society in the areas of work and social inclusion [51].

It has also been shown that previous experiences with individuals with disabilities can exert a positive influence on attitudes toward individuals with disabilities [52].

This is most significant in the area of feelings. A statistically significant association was shown for all three types of encounters. This effect is then perceived as positive. Often, it is a fear of the unknown that stems from a lack of information and can persist into adulthood and have a negative effect on professional life [53]. It is interesting to note that when questions were asked about the type of disability in friends, the following types of disability did not appear once: autism spectrum disorder, attention deficit hyperactivity disorder, and specific learning disability. These are the types of disabilities that are perhaps the most challenging in terms of relationships as a result of the difficulty in communication and the competencies needed to engage in activities together, particularly play. In this context, it is worth noting that the responses to the questions on personal experiences with individuals with disabilities revealed that 79% of the respondents had no family members with disabilities and 67% had no friends with disabilities. This is understandable as nowadays individuals with disabilities naturally appear in society and participate fully in different areas of life. The phenomenon of “friend” versus contact is interesting. From the above data on inclusive education, it is more than evident that almost every school classroom has at least one pupil with SEN. However, here the fact that most respondents probably do not perceive them as friends, or perceive as friends only those with milder types of disabilities, becomes apparent. This corresponds with the finding above, where only 21% of the respondents perceive joint education as appropriate.

Five basic codes emerged during the open coding assessment of the free responses and interviews: pity, admiration, taboo, inclusion and segregation, and differentiation. The open-ended responses revealed that approximately 20 respondents indicated that they felt tremendous regret when they encountered individuals with disabilities. Approximately 20 respondents said they found the topic unpleasant and painful and did not want to talk about it. In one case, we were even warned that this was not an appropriate topic and that the questions might cause hurt. In two cases, there was admiration. Even though this only occurred twice, a separate category was created from these responses. We perceived a strong emphasis on this view from these respondents. The last two categories are intertwined. Attitudes toward the disabled are driven by a variety of factors. Pity can be triggered by compassion for a person who is struggling with difficulties and limitations. Admiration may arise from respect for a person who is able to overcome his or her difficulties and achieve success. Taboo can be associated with negative stereotypes and prejudices against persons with disabilities. Inclusion is associated with an attitude where persons with disabilities are fully integrated into society without any discrimination or segregation. Segregation, on the other hand, is a view in which persons with disabilities are separated from the rest of society and are subjected to various types of discrimination. Differentiation can be linked to the provision of special services and support for people with disabilities to enable them to reach their full abilities and potential. It is important to engage in dialogue and seek solutions that lead to better inclusion of persons with disabilities in society without any restrictions. The results of the research presented here show the main categories that make up attitudes toward persons with disabilities and also the themes that can be used for possible intervention. Importantly, for about 150 respondents, their attitudes would depend on the type of disability. These respondents reported that their greatest fear was coming into contact with individuals with intellectual disabilities. They stated that they did not know how to treat them or were embarrassed about not understanding their reactions. The ten interviews revealed the same. The respondents reported that they stood by their general attitudes toward individuals with disabilities, but that they would change their attitudes a little in the case of individuals with intellectual disabilities: “that I am afraid of people with disabilities… I imagined people with intellectual disabilities and I didn’t think about, for example, physical disabilities as a result of an accident. I wouldn’t be so worried there… that different types of disability cannot be put on the same level in an evaluation…”. Prior to the actual research, we considered whether to construct the questionnaire to measure general attitudes or to separate attitudes and different types of disability. Given the current talk of an inclusive society, we decided to construct one on general attitudes [54]. In line with the inclusive concept, no distinctions should be made on the basis of the type of disability. Even so, some adolescents do not perceive these attitudes in the same way.

## 6. What Is the Knowledge of Adolescents about Individuals with Disabilities?

The results presented show that almost 80% of the respondents have a clear basic knowledge of individuals with disabilities. Only 15 individuals (0.8%) did not assign even one answer correctly. Good knowledge then leads to positive attitudes toward individuals with disabilities and vice versa. In the free response question and follow-up interviews, it was found that most information about individuals with disabilities is obtained by young people at school and from the media (the internet, social networks), as well as in the family or through personal contact with an individual with a disability.

The application of the research results has an impact on education. On the basis of the findings, it is possible to develop and strengthen educational programs aimed at educating and informing teenagers about persons with disabilities. These educational measures can help to reduce prejudice, stereotypes, and discrimination. The area of prevention is also important. On the basis of the facts that were established, it is possible to propose prevention aimed at strengthening positive attitudes and openness to discussing individuals with disabilities. Prevention may include workshops, presentations, or discussion groups that allow adolescents to better understand and respect the needs of individuals with disabilities. Skills and their development could also provide support for adolescents to feel comfortable interacting with individuals with disabilities, especially intellectual disabilities. This may include creating a friendly environment in which questions and discussions about disability are open and providing information on how to communicate and treat individuals with different types of disabilities. Education should also include training in empathy and understanding. Adolescents should be encouraged to understand the barriers and challenges faced by people with disabilities and how they can contribute to creating an inclusive and respectful environment. A significant role is also played by the involvement of adolescents in community activities or volunteer projects where they can meet individuals with disabilities. This interaction could help raise their awareness, change attitudes, and provide them with an opportunity to build personal relationships with these individuals. One of the findings is the difference in the attitudes of boys and girls. The findings indicate that boys, compared to girls, have fewer positive attitudes toward individuals with disabilities and are less open to discussing the topic. Therefore, special programs or initiatives aimed at supporting the positive attitudes of boys and their openness toward communication and interaction with individuals with disabilities should be targeted. Overall, these findings should be used to create an inclusive environment that embraces and respects diversity.

## 7. For Further Research

Follow-up research activities should focus on the analysis and detailed description of attitude components such as awareness, behavior, and emotional adjustment of adolescents. It would also be advisable to conduct research using the same methodology in other age groups to gain cross-sectional results with information on the development of attitudes toward the disabled in the non-disabled population to gain a deeper understanding of the issue. Another task would be to analyze curriculum documents at all levels of the educational system of the Czech Republic. By analyzing the texts, it would be possible to name opportunities suitable for educating or informing about persons with disabilities.

## 8. Conclusions

The research has shown that more than 70% of the adolescents aged 11 to 16 who were surveyed have basic knowledge of individuals with disabilities. Further, these adolescents were shown to have an overall positive attitude toward individuals with disabilities. This relationship is statistically significantly better for the girls than the boys, and the girls are also more open to discussing individuals with disabilities. At the same time, even when attitudes are positive, they still depend on the type of disability. The greatest insecurity or discomfort is shown when interacting with individuals with intellectual disabilities. Furthermore, positive attitudes appeared to be directed toward social functioning but not toward learning together. Here, rather segregationist attitudes became clear. For future research, it would be interesting to focus on attitudes toward specific types of disability. The results obtained could then be reflected in the educational process.

In today’s inclusive society, it is crucial to foster relationships between non-disabled children and their disabled classmates. These relationships promote empathy and understanding and create an environment where all students feel valued and included. To achieve this, several strategies can be implemented. Firstly, it is essential to educate non-disabled children about disabilities. As a result of their awareness being increased, children will better understand the challenges their disabled classmates face and also their unique abilities. Group projects or classroom activities encouraging collaboration among students with diverse skills can help break down barriers and build friendships. By highlighting each student’s strengths, regardless of ability, a sense of belonging will be cultivated among all classmates. Promoting peer support programs can significantly enhance relationships between non-disabled children and their disabled classmates. Pairing students together for mentoring or buddy systems allows for regular interaction outside the classroom, fostering genuine connections based on mutual trust and support. Another option is the intervention of a special educator or psychologist to help correct the relevant deficits.

## 9. Implication and Contribution to Further Research

Subsequent research activities should be directed toward the analysis and detailed description of components of attitudes such as awareness, behavior, and emotional adjustment.

## 10. Study Limits

A random sampling method was used to select the sample, but it does not reflect the total population of the age group under study. This results in the limitation of the study through ”sampling bias”. To obtain more accurate results, it would be desirable to focus more on a larger representation of schools by type of study—vocational training schools and schools preparing for further study. Overall, a larger number of respondents always leads to more conclusive results. The study was based on “volunteering” to work with the researchers.

## Figures and Tables

**Table 1:** Distribution of respondents according to age and gender.

	Age						Gender		
	11	12	13	14	15	16	Girls	Boys	Not Specified
Number	179	457	463	409	272	26	880	912	14
Percent	10%	25%	26%	23%	15%	1%	49%	50%	1%

**Table 2:** Questions exploring the respondents’ experiences with persons with disabilities in their own family and social environment.

Question	Yes	No	I Don’t Know
If there was an individual with a disability in the family	346 (19%)	1435 (79%)	25 (2%)
If they had a friend with a disability	544 (30%)	1214 (67%)	48 (3%)
If the respondents had ever met an individual with a disability	1360 (75%)	259 (14%)	187 (11%)

**Table 3:** Frequencies and percentages of questions (7–9, 12, 14, and18).

	7		8		9		12		14		18	
	Frequency	%	Frequency	%	Frequency	%	Frequency	%	Frequency	%	Frequency	%
1	180	10.0	97	5.4	235	13.0	167	9.2	230	12.7	166	9.2
2	410	22.7	362	20.0	456	25.2	308	17.1	397	22.0	219	12.1
3	708	39.2	714	39.5	600	33.2	725	40.1	607	33.6	752	41.6
4	333	18.4	416	23.0	303	16.8	420	23.3	418	23.1	376	20.8
5	175	9.7	217	12.0	212	11.7	186	10.3	154	8.5	293	16.2
Total	1806	100.0	1806	100.0	235	13.0	1806	100.0	1806	100.0	1806	100.0

**Table 4:** Frequencies and percentages of questions (10–11 and 15–17).

		10		11		15		16		17	
		Frequency	%	Frequency	%	Frequency	%	Frequency	%	Frequency	%
Valid	1	396	21.9	578	32.0	904	50.1	704	39.0	446	24.7
	2	448	24.8	684	37.9	487	27.0	585	32.4	443	24.5
	3	638	35.3	366	20.3	267	14.8	389	21.5	571	31.6
	4	185	10.2	124	6.9	86	4.8	75	4.2	227	12.6
	5	139	7.7	54	3.0	62	3.4	53	2.9	119	6.6
	Total	1806	100.0	1806	100.0	1806	100.0	1806	100.0	1806	100.0

**Table 5:** Factors extracted and their saturation.

Factor	Items	Factorial Saturations
Acceptance	12	0.69
	13	0.63
	15	0.56
	17	0.53
	14	0.48
	16	0.42
Feelings	7	0.72
	9	0.58
	8	0.55
	10	0.54
Competence	18	0.78
	11	0.62

**Table 6:** Correlation between personal experience and attitudes.

Previous Contact	Correlation
Individual with a disability in the family	r = −0.09, *n* = 1806, *p* < 0.001
Friend with a disability	r = −0.09, *n* = 1806, *p* < 0.001
Previous contact with an individual with a disability	r = −0.12, *n* = 1806, *p* < 0.001

**Table 7:** Previous personal contact.

	Correlation Acceptance	Correlation Feelings	Correlation Competence
Individual with a disability in the family	NS	r = −0.09, *n* = 1806, *p* < 0.001	r = −0.07, *n* = 1806, *p* = 0.003
Friend with a disability	r = −0.07, *n* = 1806, *p* = 0.006	r = −0.12, *n* = 1806, *p* < 0.001	NS
Previous contact with an individual with a disability	r = −0.12, *n* = 1806, *p* < 0.001	r = −0.12, *n* = 1806, *p* < 0.001	NS

## Data Availability

The data presented in this study are available on request from the corresponding author. The data are not publicly available for privacy reasons.
